# Mechanistic insights into silica nanoparticle–allergen interactions on antigen presenting cell function in the context of allergic reactions†

**DOI:** 10.1039/d2nr05181h

**Published:** 2023-01-11

**Authors:** Litty Johnson, Lorenz Aglas, Benjamin Punz, Hieu-Hoa Dang, Constantin Christ, Lisa Pointner, Mario Wenger, Norbert Hofstaetter, Sabine Hofer, Mark Geppert, Ancuela Andosch, Fatima Ferreira, Jutta Horejs-Hoeck, Albert Duschl, Martin Himly

**Affiliations:** a Department of Biosciences and Medical Biology, University of Salzburg 5020 Salzburg Austria martin.himly@plus.ac.at

## Abstract

The incorporation of nanomaterials into consumer products has substantially increased in recent years, raising concerns about their safety. The inherent physicochemical properties of nanoparticles allow them to cross epithelial barriers and gain access to immunocompetent cells. Nanoparticles in cosmetic products can potentially interact with environmental allergens, forming a protein corona, and together penetrate through damaged skin. Allergen–nanoparticle interactions may influence the immune response, eventually resulting in an adverse or beneficial outcome in terms of allergic reactivity. This study determines the impact of silica nanoparticle–allergen interactions on allergic sensitization by studying the major molecular mechanisms affecting allergic responses. The major birch pollen allergen Bet v 1 was chosen as a model allergen and the birch pollen extract as a comparator. Key events in immunotoxicity including allergen uptake, processing, presentation, expression of costimulatory molecules and cytokine release were studied in human monocyte-derived dendritic cells. Using an *in vivo* sensitization model, murine Bet v 1-specific IgG and IgE levels were monitored. Upon the interaction of allergens with silica nanoparticles, we observed an enhanced uptake of the allergen by macropinocytosis, improved proteolytic processing, and presentation concomitant with a propensity to increase allergen-specific IgG2a and decrease IgE antibody levels. Together, these events suggest that upon nanoparticle interactions the immune response is biased towards a type 1 inflammatory profile, characterized by the upregulation of T helper 1 (Th1) cells. In conclusion, the interaction of the birch pollen allergen with silica nanoparticles will not worsen allergic sensitization, a state of type 2-inflammation, but rather seems to decrease it by skewing towards a Th1-dominated immune response.

## Abstract 2

Impact of SiO_2 _NP-allergen interaction on dendritic cell function altering the immune response, eventually resulting in a harmless, beneficial outcome in terms of allergic reactivity.

## Graphical abstract

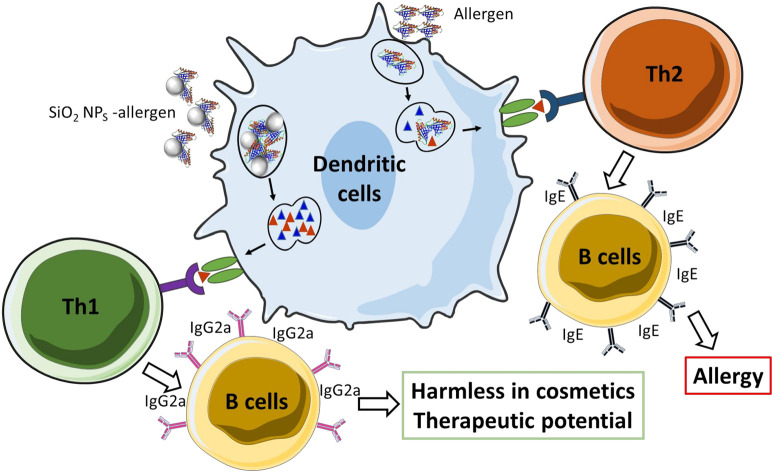

## Introduction

The primary function of the adaptive immune system is host defence against microbes; however, some immune responses are directed against harmless antigens, such as in the case of allergic or atopic diseases.^1^ Allergic diseases are increasing worldwide and are considered, for example, in Europe as the most common chronic disease with a negative impact on quality of life.^2^ Allergic diseases develop *via* a sensitization phase (primary immune response to an antigen) followed by effector (secondary immune response) phases, where the exposure to allergen elicits symptoms including allergic rhinitis, allergic asthma, atopic dermatitis, atopic eczema, atopic urticaria, or systemic reactions (anaphylaxis). Nanoparticles can play three different roles in the context of allergic diseases.

First, they may have the potential to aggravate the immunological response to an allergen, worsening the clinical conditions.^3^ For example, Chuang *et al.* demonstrated that the inhalation of 33 nm silver nanoparticles, followed by ovalbumin (allergen) challenge led to the induction of an allergic response with exacerbated allergic asthma in healthy and allergic mice.^4^ Intranasal/subcutaneous administration of carbon nanotubes (CNTs) with ovalbumin in mice increased the amount of allergen-specific IgE antibodies in the blood and the eosinophil counts in the BALF, and elevated the secretion of Th2-associated cytokines.^5^ Amorphous silica nanoparticles with the house dust mite (*Dermatophagoides pteronyssinus*) antigen injected intradermally into mice caused a size-dependent exacerbation of atopic dermatitis indicated by increased serum IgE levels, Th2 cytokine release (IL-4) and cytokines associated with atopic dermatitis (IL-18, TSLP).^6^ Agglomerates of silica nanoparticles and *D. pteronyssinus* extract, when administered topically to mice, elicited an IgE-biased immune response with enhanced vulnerability to anaphylaxis.^7^ Ives *et al.* showed that allergic mice produced more IgE antibodies when exposed to zinc oxide nanoparticles through their skin.^8^ Taken together, these studies showed that exposing animals simultaneously to nanomaterials and allergens can enhance the allergic responses, although the underlying molecular mechanisms remained mainly unknown and the models used were not designed to mimic actual human exposure situations.

Second, nanoparticles are investigated as adjuvants or/and carriers in allergen-specific immunotherapy (AIT), which is so far the only curative treatment for allergy.^9^ The high dose of an allergen that is required for the induction of tolerance in AIT can be loaded efficiently onto the enormous surface area of nanoparticles. Furthermore, NPs can promote immunomodulation especially towards a type 1 or tolerogenic immune response, which is the desired goal in AIT. Numerous nanoparticles, including virus-like particles,^10^ liposomes,^11^ and polymeric nanoparticles,^12^ have shown promising results for application in AIT. However, more candidate nanoparticles need to be investigated to develop improved therapeutic strategies with better safety and efficacy profiles for the treatment of allergies.^13^ By shortening the length of the therapy and lowering the cost of care, nanoparticles would have the potential to promote patient compliance in allergy treatment.^14^ To explore further on their action as adjuvants, in particular for improving their efficacy, it will be crucial to understand the immunomodulatory characteristics of nanomaterials and the underlying molecular mechanisms.^9^

Third, nanoparticles may contribute to allergic sensitization indirectly as bystanders, and potentially lead even to increased incidence of allergies by different mechanisms involving the combination with other substances. Nanomaterials in cosmetics can interact with emulsifiers, surfactants and other components, which may cause disruption of epithelial barriers and microbial dysbiosis, recognized to be causal for allergic diseases.^15^ Notably, some allergens derived from house dust mite and pollen are capable of disrupting the integrity of the epithelial barrier due to their inherent protease activity^16^ and can cause allergic sensitization through the skin.^17,18^ Nanomaterials improve skin penetration and rehydration by optimizing the entrapment of active ingredients^19^ and may penetrate both healthy and injured skin.^20^ It has to be considered that due to the nature of the allergic process, even a very low exposure could be sufficient for sensitization. In general, when allergenic proteins bind to nanoparticles, this can induce structural alterations.^21,22^ In addition, they may bind in a non-randomized manner and, thus, selectively hide or expose allergenic epitopes on their surface.^23,24^ Consequently, their biological effects may change, increasing or decreasing the immune response towards them. Therefore, nanoparticle–allergen conjugates could change the immunological effects of allergens. The impact of nanoparticle–allergen interactions during allergenic sensitization, when symptoms are not yet established, is not well understood.

In this study we aimed to examine the impact of nanoparticle–allergen interactions on the initiation of an allergic response by studying the cellular and molecular key events driving it. Amorphous silica nanoparticles (SiO_2_ NPs), which are widely employed in the cosmetics sector, were adopted for the study. They serve as an adsorbent, anti-caking, dispersing/suspending, and bulking agent and aid in increasing the flow properties and adherence in cosmetics.^25^ The European Union has authorized SiO_2_ NPs for use in cosmetics and personal care products.^26^ The major birch pollen allergen (Bet v 1.0101) and birch pollen extract were used as model allergens, as the prevalence of respiratory allergies induced by pollen is still increasing in Europe (estimated now to be more than 40%).^27^ As Bet v 1 by itself has been reported to be immunologically inert,^28^ the chosen approach would help in dissecting the impact of nanoparticle interactions from bystander effects, derived, for instance, from endotoxin present in the environmental allergens. The initiation and control of an immune response to allergens largely depend on the action of antigen-presenting cells (APCs). Among the professional APCs, dendritic cells (DCs) play a primary role in the sensitization phase of allergy.^29^ Monocyte-derived dendritic cells (moDCs) are a widely used model for studying the function of APCs, and exert crucial immune functions in the mucosal, epithelial and dermal tissues of the lungs, gut and skin.^30–32^ We thus explored the impact of nanoparticle–allergen interaction on DC function by investigating the primary key events related to allergic sensitization in moDCs. A greater knowledge of these underlying mechanisms may also contribute to the development of innovative immunotherapy approaches using nanoparticles.

## Results and discussion

### SiO_2_ NP and allergen conjugation

The SiO_2_ NP suspension used for the study was well dispersed with a hydrodynamic particle size of 100.3 ± 3.4 nm (a polydispersity index of 0.025) and exhibited a zeta potential of −38.9 ± 2.8 mV (Table S2†). The primary particle size, as determined by TEM, was 96.3 ± 4.9 nm (Fig. S1†). The allergens were conjugated to the nanoparticles by non-covalent adsorption in an isotonic environment by maintaining a pH of 7.4. The detailed production and characterization of the particles together with the conjugation efficiency can be found in a previous report and ESI Fig. S1 and Table S2.†^21^

**Fig. 1:**
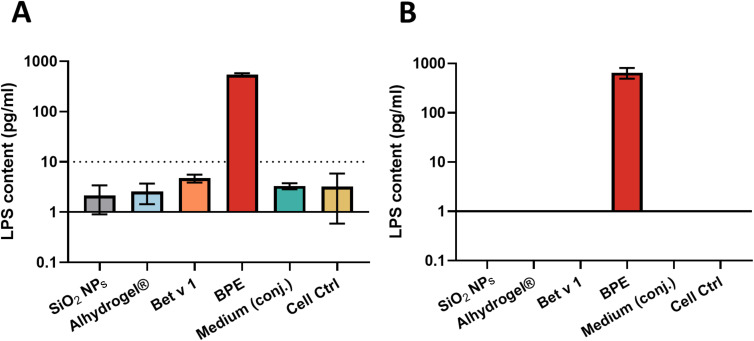
Endotoxin/LPS contamination levels on the particles, allergen, allergen extract and medium measured by (A) the HEK BlueTM hTLR4 assay and (B) monocyte activation test. The LPS contents were determined in the assays based on a standard LPS curve from *E. coli* (1500 pg ml^−1^ to 1 pg ml^−1^). The LPS content represented in the monocyte activation test is the average of LPS concentration measured in both IL-6 and TNF-α ELISA. Endotoxin-free water, represented as “Cell Ctrl” was used as the negative control. SiO_2_ NPs (100 μg ml^−1^), Alhydrogel® (100 μg ml^−1^), Bet v 1 (10 μg ml^−1^) and BPE (100 μg ml^−1^) were used for testing the endotoxin content.

Endotoxin or lipopolysaccharide (LPS) from Gram-negative microorganisms harbors immunostimulatory capabilities, activating primary human immune cells by triggering toll-like receptors. In general, moDCs can be activated by endotoxin concentrations as low as 20 pg ml^−1^.^33^ Therefore, the endotoxin content on the particles, allergen, and medium was examined using the HEK Blue™ human toll-like receptor 4 (hTLR4) assay and the monocyte activation test to confirm that the effects of particle stimulation on DCs were not caused by endotoxin contamination. The LPS content in the samples was determined by comparing the NF-κB and monocyte activation induced by LPS with a standard curve. For SiO_2_ NPs, Alhydrogel®, Bet v 1.0101 (represented as Bet v 1) and conjugation medium, the HEK Blue™ hTLR4 assay showed a level of less than 10 pg ml^−1^ and was undetectable in MAT. However, as anticipated, the birch pollen extract (BPE) showed around 600 pg ml^−1^ of LPS contamination in both the assays (Fig. 1A and B).

### Enhanced cellular delivery of the allergen mediated by macropinocytosis

**Fig. 2:**
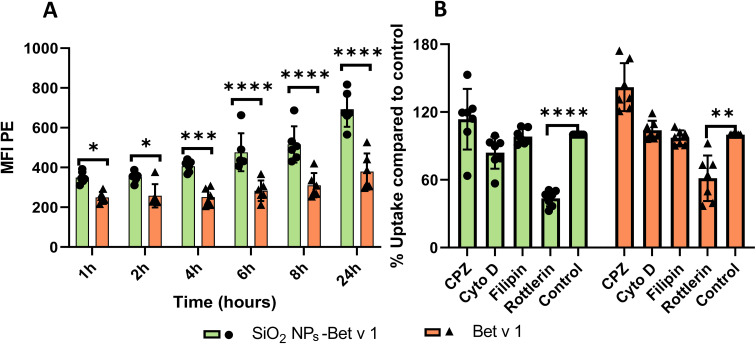
SiO_2_ NPs facilitate the endocytosis of allergen *via* macropinocytosis. (A) Kinetics of internalization of Bet v 1 by moDCs on exposure to the SiO_2_ NP–allergen conjugates. (B) Mechanism of the uptake of Bet v 1 with and without conjugation with SiO_2_ NPs in moDCs after 24 hours stimulation determined by employing different endocytosis inhibitors. Data are shown as the means ± SD of at least cells from 5 individual donors. Results shown are the representative of more than three independent experiments. Statistical analysis was performed using repeated-measures ANOVA with the Bonferroni correction. ****P < 0.0001, *** *P* < 0.0002, **P < 0.002, *P < 0.03. MFI PE, mean fluorescence intensity within a phycoerythrin channel (flow cytometry).

**Fig. 3:**
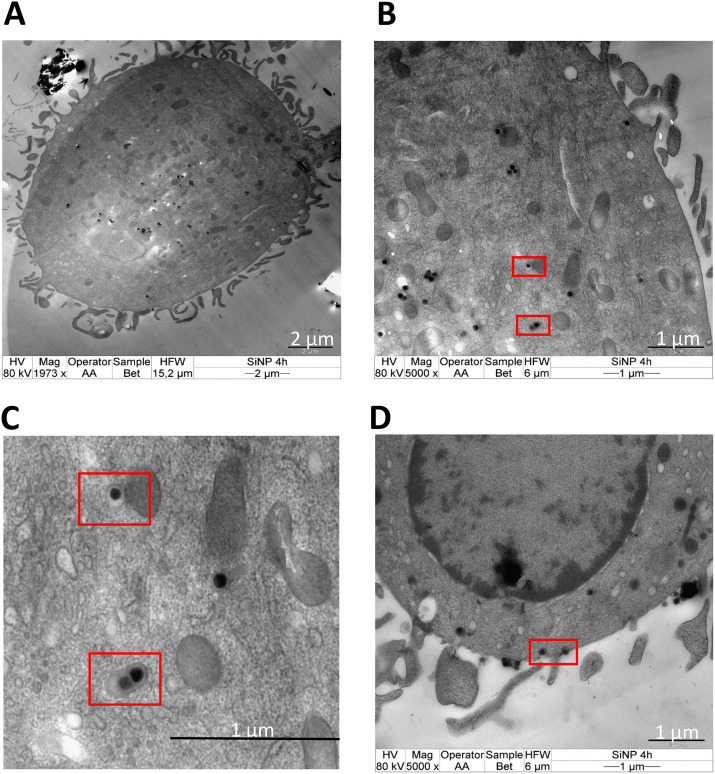
Macropinocytosis-mediated uptake and intracellular localization of SiO_2_ NPs by moDCs. TEM images of moDCs showing (A) the overall localization of SiO_2_ NPs, (B) internalization of SiO_2_ NPs in the macropinosome, (C) zoomed in at representative macropinosomes and (D) membrane ruffle formation during macropinocytosis after 4 hours of stimulation.

APCs play a crucial role in initiating the early events in the humoral and cellular responses in allergy.^29^ The first stage in the sensitization of allergen after its trans-epithelial passage involves its internalization by APCs. The nature of allergen, together with the internalization process, can influence allergic sensitization.^29,34^ We, thus, determined the differences in the endocytosis of allergen by moDCs when conjugated to nanoparticles. Initially, the stability of nanoparticle–allergen conjugates in suspension was tested by the blue silicomolybdic assay to ensure that the delivered dose was similar to the administered dose (Fig. S2†). The kinetics and mechanism of uptake were then studied by flow cytometry where the fluorescence intensity of the labelled Bet v 1 (pHrodo succinimidyl ester) with/without association of SiO_2_ NPs was measured in moDCs at different time points. The results revealed increased fluorescence intensity for SiO_2_ NPs-Bet v 1 at all investigated timepoints (Fig. 2A), indicating an enhanced cellular internalization of Bet v 1 when associated with nanoparticles. However, the investigation of the uptake mechanism using inhibitors of major endocytosis mechanisms (Table S1†) revealed no differences between the conjugated and unconjugated Bet v 1. The endocytosis inhibitors had no impact on the viability of moDCs (Fig. S3†). moDCs internalized the conjugated (SiO_2_ NPs-Bet v 1) and unconjugated Bet v 1 mainly by macropinocytosis as indicated by the decreased mean fluorescence intensity (MFI) using rottlerin (a selective inhibitor for macropinocytosis) (Fig. 2B). This perfectly matches with the previously published reports where Noirey *et al.* and Smole *et al.* demonstrated that macropinocytosis is the major mechanism driving the endocytosis of Bet v 1.^35,36^ Furthermore, the visual observation of the stimulated moDCs by transmission electron microscopy (TEM) showed the uptake of SiO_2_ NPs into the cells (Fig. 3A) upon the engulfment of nanoparticle conjugates in the intracellular macropinosomes (Fig. 3B and C) due to the formation of membrane ruffles (Fig. 3D). These data, together, demonstrate that the interaction of SiO_2_ NPs with Bet v 1 enhances the endocytosis of allergen by macropinocytosis.

For both, understanding their cytotoxic effects and for the biomedical use of nanoparticles, it is essential to confirm their cellular fate when they enter the cells. Hence, an indirect technique was used to assess the exocytosis of nanoparticles by moDCs. moDCs were stimulated with the same volume/concentration of conjugates for various time periods, carefully washing with PBS to remove the non-internalized nanoparticles, and the amount of silica inside the cells was calculated. Interestingly, the concentration of silica gradually increased until 4 hours, followed by a decrease from 6 to 24 hours (Fig. S4†). The decrease in the silica concentration would not be due to the dissolution of SiO_2_ NPs as the dissolution of silica in the acidic environment inside the cells is reported to be negligible.^37^ This evidences a marked and efficient uptake followed by gradual exocytosis of nanoparticles from the cells after the internalization. The efficient internalization of allergen is accomplished by the large surface area of nanoparticles that increases the allergen interaction.^38^ A high dose of allergen has been shown to elicit differentiation towards regulatory T cells (Treg) rather than Th2 differentiation.^39,40^ This is the desired immune response in AIT. The enhanced internalization of allergen observed here has the potential to induce allergen tolerance, which is the hallmark of a healthy immune response to the allergen. This indicates that the nanoparticle–allergen interaction may not only inhibit allergic sensitization but also enhance the therapeutic efficacy in AIT. Additionally, exocytosis of nanoparticles is crucial to prevent any potential cell toxicity and to speed up their clearance from the body. There have been reports that 50 nm silica nanoparticles are promptly exocytosed from cancer cell lines, balancing the number of nanoparticles inside and outside the cells.^41^ Experimental evidence suggests that SiO_2_ NPs of approximately 100 nm size are excreted in the bile and urine.^42^ Altogether, the accelerated endocytosis of allergen followed by the exocytosis of SiO_2_ NPs supports their potential application as an efficient carrier for AIT. Furthermore, the application of SiO_2_ NPs in cosmetics potentially enabling an interaction with the allergen *in situ* may result in a beneficial rather than an adverse outcome.

### Nanoparticle interaction boosts the proteolytic processing and presentation of allergens

**Fig. 4:**
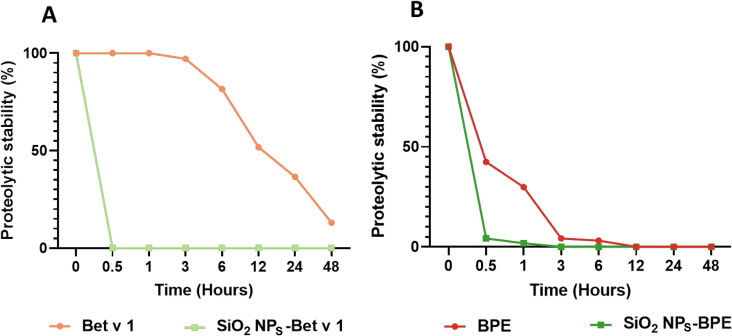
SiO_2_ NP interaction enhances the proteolytic processing of allergen. The kinetics of the proteolytic degradation of SiO_2_ NPs conjugated to (A) Bet v 1 (B) BPE assessed by gel electrophoresis using recombinant cathepsin S for simulating degradation in APCs. Proteolytic stability values were determined based on the intensity of intact protein bands compared to calibrator bands using Image Lab 4.01 software (Bio-Rad, Hercules, California, USA).

Allergens internalized by the APCs are cleaved into their peptide fragments in the endolysosomal compartment by proteases like cathepsin S. The so-formed peptides were then loaded onto the class II MHC molecules (MHC II) and displayed on the cell surface, facilitating the activation of naïve T cells and tailoring the subsequent immune response. The efficiency of endolysosomal allergen processing by DCs can impact allergic sensitization by modifying the quality of the subsequent T cell response.^43^ We, thus, determined if the interaction with SiO_2_ NPs affected the proteolytic processing of Bet v 1 using recombinant cathepsin S, an endolysosomal cysteine protease that plays an integral role in the degradation of protein antigens in APCs.^44^ A significant increase in the proteolytic degradation of SiO_2_ NPs-Bet v 1 was observed when compared to the unconjugated allergen (Fig. 4A). Conjugation of Bet v 1 to SiO_2_ NPs resulted in the complete degradation of allergen within 30 minutes; however, the unconjugated allergen was rather stable (50%) even after 12 hours. Similar degradation patterns were observed with both the unconjugated and conjugated Bet v 1 using the endolysosomal degradation assay (Fig. S5†). These results are described in detail in our previously reported paper.^21^ Additionally we observed the generation of more peptides, especially, in the immunodominant T cell-activating region. The degradation kinetics of Bet v 1 were similar to previously reported results.^45,46^ Even though natural Bet v 1 and recombinant Bet v 1 have shown negligible differences in their immune activation profiles,^28^ we wanted to see if the conjugation of birch pollen extract (BPE) to SiO_2_ NPs altered the proteolytic degradation kinetics of natural Bet v 1. Similar to recombinant Bet v 1, natural Bet v 1 in the BPE showed an enhancement in proteolytic degradation when associated with SiO_2_ NPs (Fig. 4B). Non-covalent interactions have been reported to impact the structural stability of the antigen, altering the efficacy of antigen presentation.^47^ We have previously shown that the interaction of allergens with nanoparticles changes a 3D fold of the allergen which is a determinant for proteolytic stability. The non-covalent adsorption of Bet v 1 to SiO_2_ NPs distorted the alpha-helical structure of the allergen.^21^ This structural alteration induced by nanoparticles changes the conformational stability of the allergen, ultimately decreasing their proteolytic stability. Altogether, the interaction of nanoparticles accelerated the processing of allergens in the endolysosomal compartments and subsequently increased the peptide generation.

**Fig. 5:**
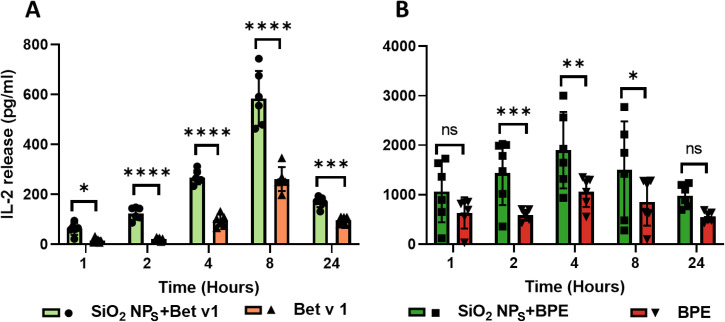
SiO_2_ NPs enhance the activation of naïve T cells upon the presentation of allergen by APCs. IL-2 secretion from T cell hybridoma cells was used as the read out for T cell activation upon the presentation of immunodominant peptides (142–153) by BMDCs at different time points for (A) Bet v 1 and (B) the BPE. Data are shown as the means ± SD from 6 individual experiments. Statistical analysis was performed using ordinary two-way ANOVA with the Bonferroni correction. ****P < 0.0001, ***P < 0.0002, **P < 0.002, *P < 0.03.

The enhanced proteolytic processing of allergen can result in an increased presentation of allergenic peptides to CD4^+^, *i.e.*, helper T cells. However, if the generated peptides have less binding affinity to MHC II molecules, the surface display of peptide-MHC II complexes decreases, affecting antigen presentation.^48^ Although we observed an increased generation of peptides in the immunodominant T cell epitope region, nanoparticle interaction might have altered the length of the generated peptide, changing the binding affinity. An optimal length of the peptide (about 18 to 20 amino acids) is desirable for MHC II binding.^49^ Thus, to assess if the nanoparticle interaction affected the allergen presentation, T cell hybridoma cells specific for the dominant T cell epitope were co-cultured with mouse bone marrow-derived DCs (BMDCs) that had previously been stimulated with the conjugated *vs.* unconjugated allergen, and the extent of T cell activation was measured by the release of IL-2. SiO_2_ NPs-Bet v 1 showed a significant increase in IL-2 production compared to Bet v 1 at all the examined time points (Fig. 5A). A similar increase was also observed for SiO_2_ NPs-BPE in comparison with the BPE, except at 1 and 24 hours where rather equal amounts were measured (Fig. 5B). SiO_2_ NPs by themselves did not lead to a significant release of IL-2, which was found to be similar to that of the cell control (Fig. S6†). Overall, the binding affinity to MHC II molecules was not altered by the peptides produced by the endolysosomal degradation of SiO_2_ NPs-Bet v 1/BPE. Additionally, the increased peptide production led to an improved peptide-MHC II complex display, enhancing antigen presentation and thus T cell receptor (TCR) signalling.

Altered processing of allergen can affect its immunogenicity and hence modulate immune polarization. It has been demonstrated that inefficient processing of the stabilized Bet v 1 variants resulted in decreased peptide/MHC densities favouring Th2 polarization.^50^ An enhanced processing of allergen generating higher peptide densities was stated to predominantly favor a Th1-dominated immune response.^43^ Furthermore, the decreased proteolytic stability can favor a protective immune response as the immunogenicity of allergen was reduced due to the loss of conformational epitopes.^43^ However, we found that SiO_2_ NPs increased TCR signalling, demonstrating the integrity of the allergen's T cell epitopes. Strong TCR signalling has been demonstrated to preferentially produce a Th1 polarization, whereas weak TCR signalling favors the generation of Th2 cells.^51^ Adjuvants for AIT including CpG oligodeoxynucleotide (CPG ODN) or monophosphoryl lipid A (MPL) have been reported to skew the immune response towards Th1-dominated responses.^52^ When these reports are compared to our findings, we may infer that the interaction of SiO_2_ NPs with an allergen triggers a protective immune response that is directed towards the Th1-driven response, suppressing allergic sensitization and demonstrating the potential application of SiO_2_ NPs as adjuvants in AIT. In addition, SiO_2_ NPs in cosmetic products may have a positive rather than a negative consequence in the context of allergic sensitization. However, the co-stimulatory molecules and cytokine milieu, in addition to TCR signalling, play a significant role in promoting immunological polarization and thus were investigated next.

### Negligible impact on the maturation of moDCs by nanoparticle–allergen interactions

**Fig. 6:**
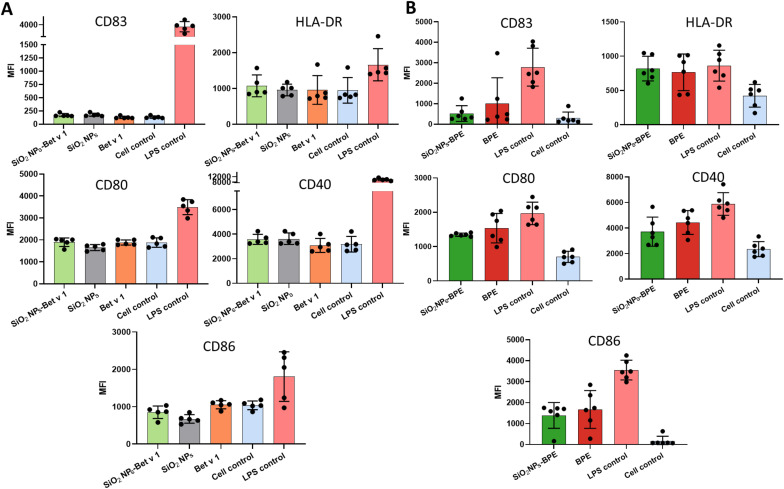
The analysis of the maturation of moDCs measured by surface marker expression. moDCs were incubated with (A) Bet v 1 (conjugated and unconjugated) (B) the BPE (conjugated and unconjugated). The levels of CD83, CD40, CD80, CD86 and HLA-DR were assessed by flow cytometry. Data are presented as the mean fluorescence intensity (MFI) and are the means ± SD of at least cells from 5 individual donors (for Bet v 1) and 6 individual donors (for BPE). Results shown are the representative of more than three independent experiments. Statistical analysis was performed using repeated measures ANOVA with Tukey's *post-hoc* test. LPS control (100 ng ml^−1^) was excluded from the statistical analysis.

Dendritic cells undergo maturation in response to an antigen to facilitate the proliferation and differentiation of effector T cells. The maturation in DCs is characterized by the upregulation of co-stimulatory molecules (CD80, CD83, CD86, and CD40), major histocompatibility complex (MHC) molecules at the cell surface and the release of cytokines. The co-stimulatory molecules together with the cytokine microenvironment play a critical role in eliciting the T cell immune response (Th1/Th2) and maintaining T cell tolerance. Hence, we determined if the interaction of nanoparticles with the allergen is altering the maturation status of DCs, thereby affecting allergic sensitization. Thus, we stimulated moDCs with the samples for 24 hours and measured their surface markers as well as the cytokine expression profile using a 45-plex array. There were no significant differences in the level of DC maturation markers including CD80, CD83, CD86, CD40 and HLA-DR in SiO_2_ NPs-Bet v 1 when compared to unconjugated Bet v 1 (Fig. 6A). From our observation, Bet v 1 was evidenced to be inefficient in activating bone marrow-derived DCs (BMDCs) compared to the BPE,^28^ exploring for a generally immune-inert behaviour of highly purified endotoxin-free allergens in these models. Hence, we tested the maturation of moDCs using the BPE. The surface marker expression in SiO_2_ NPs-BPE was also only marginally different in this instance (Fig. 6B). However, the mean fluorescence intensity of CD86 and CD40 was markedly higher for BPE than Bet v 1, as expected, (Fig. S7†) most probably due to the presence of birch pollen-derived immunostimulatory components.^28^ Furthermore, the cytokine levels in the culture supernatants were assessed upon the stimulation of moDCs. In the case of Bet v 1 samples, we only saw quantifiable amounts of IL-1RA, IL-8, MCP-1, MIP-1α, and MIP-1β among the 45 cytokines examined, and there were no variations between Bet v 1 and SiO_2_ NPs-Bet v 1 (Fig. S8†). BPE stimulation increased additionally the release of IL-4, TNF-α and IP-10 in comparison with Bet v 1; nevertheless, no changes were detected between the BPE and SiO_2_ NPs-BPE (Fig. S9†). Altogether, the interaction of SiO_2_ NPs with the allergen did not affect the maturation state of DCs.

Nanoparticles and allergens have been previously shown to induce the maturation of DCs. For instance, galactofuranose-coated gold nanoparticles and amphiphilic poly(γ-glutamic acid) nanoparticles have been demonstrated to induce the maturation of DCs in dependence on their size and surface functionalization.^53,54^ Similarly, Der p 1, the major house dust mite allergen, and the peach allergen Pru p 3 directly induced the maturation of moDCs.^55,56^ However, there are also contradictory studies that state the opposite for both allergens^28,57^ and nanoparticles.^58,59^ Feray *et al.* reported the upregulation of maturation markers, CD86 and CD83, in moDCs when stimulated with amorphous silica nanoparticles (fumigated) of about 15 nm size.^60^ In contrast, crystalline silica inhibited the costimulatory molecules CD80, CD86 and MHC II in DCs from rat peripheral blood mononuclear cells.^61^ However, in our observation we neither saw inhibition nor stimulation in the maturation marker, as we have used colloidal amorphous silica nanoparticles of 100 nm which exhibited lower toxicity. Although the interaction with nanoparticles improved allergen uptake, proteolytic processing, and presentation, it had no effect on the maturation of DCs. In the case of SiO_2_ NPs-Bet v 1, the co-stimulatory molecule expression is comparable to that of the unstimulated cell control, hence neither T cell proliferation nor differentiation to Th1 or Th2 would ideally occur. The enhanced T cell activation through the allergen-specific TCR in the absence of co-stimulatory molecules can result in a state of T cell clonal anergy^62^ which would be beneficial in AIT. Nevertheless, it is evident from the BPE findings that SiO_2_ NPs do not suppress the production of co-stimulatory molecules, hence disproving the existence of a state of anergy. Either we may conclude from all these findings that the SiO_2_ NP–allergen interaction does not change moDC maturation directly or, as the stimulation of immature moDCs with the predominant adjuvant in AIT, aluminium hydroxide did also not result in the expression of cell surface markers,^63^ we cannot rule out the possibility that human moDCs do not represent an optimal model to study the maturation state of APCs. We, thus, went on to study their immune profile *in vivo*.

### Nanoparticle interaction modulates allergen-specific antibody responses *in vivo*

**Fig. 7:**
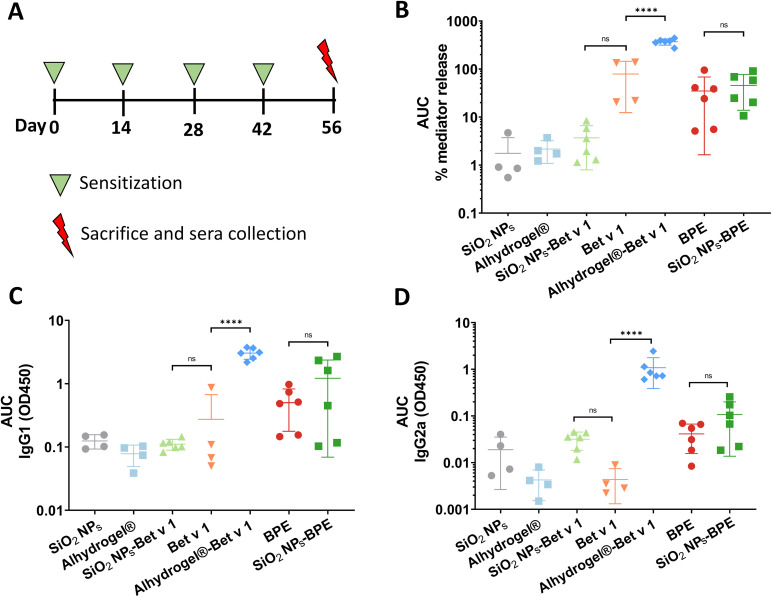
Production of allergen-specific antibody levels in a mouse sensitization model. (A) Schedule of sensitization and (B) Bet v 1-specific serum IgE post-treatment measured by muRBL assay, (C) IgG1, and (D) IgG2a measured by ELISA. Mice were sensitized by a subcutaneous injection of 6.5 μg equivalent Bet v 1 per dose and the serum from the blood was collected on the 56^th^ day, which was 14 days after the 4^th^ immunization. Unconjugated SiO_2_ NPs, Alhydrogel® and PBS were used as controls. The AUC values were calculated from Fig. S11† presenting 450 nm absorbance values *vs.* log dilutions using the trapezoid rule employing GraphPad Prism 9.4. For IgG1 serum dilutions were ranging from 200 to 102 400 and for IgG2a serum dilutions were from 50 to 1600. Data are shown as the means ± SD from at least 4 individual mice. Statistical analysis was performed using ordinary one-way ANOVA with Tukey's *post-hoc* test. ****P < 0.0001 and ns > 0.1.

**Fig. 8:**
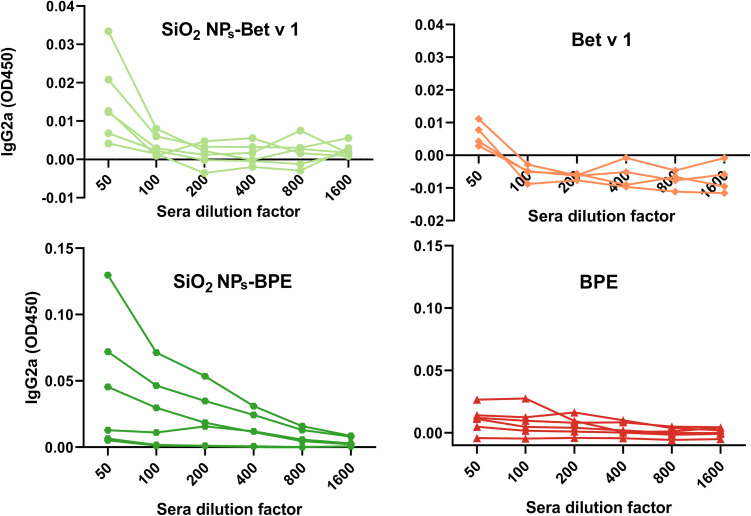
Comparison of the allergen-specific IgG2a levels of individually inspected sera from the mice. Data represent the OD450 values obtained after a serial dilution of sera from the mice treated with SiO_2_ NPs-Bet v 1 *vs.* Bet v 1 and SiO_2_ NPs-BPE *vs.* BPE.

An increased generation of allergen-specific IgE antibodies is a hallmark of allergic sensitization, and therapeutic interventions for allergic disease have been associated with high-titer blocking IgG antibody production. As the final step to evaluate the impact of the nanoparticle–allergen interaction, mice were exposed to the conjugated and unconjugated allergens through subcutaneous injections. A subcutaneous route of sensitization is considered superior for inducing allergic sensitization to Bet v 1.^64^ Alhydrogel®/alum, the most predominant adjuvant in AIT was used as a positive control for the *in vivo* experiments. The levels of functional allergen-specific antibodies were measured using the murine rat basophil leukemia (muRBL) mediator release assay (IgE) and ELISA (IgG1 and IgG2a). IgE antibody levels were indirectly quantified by the mediator release assay based on the ability of the sera of mice (exposed to samples) to induce the degranulation of muRBL cells to exhibit their genuine functionality. As anticipated, the incubation of muRBL cells with the sera of mice that received Alhydrogel®-Bet v 1 resulted in a significant increase in mediator (β-hexosaminidase) release, indicating an enhanced IgE production (Fig. 7A). The enhanced stimulation of IgE antibodies is considered as one of the unfavorable consequences of aluminium hydroxide-based adjuvants in allergy vaccination.^65^ However, the mediator release of SiO_2_ NPs-BPE and BPE did not differ from each other, and as a result, their IgE antibody levels did not differ notably (Fig. 7A). Although there were no significant differences in the mediator release between SiO_2_ NPs-Bet v 1 and Bet v 1 due to the increased variability among the different mice in the Bet v 1 group, it was evident that SiO_2_ NPs-Bet v 1 exhibits a decreasing tendency in mediator release when we look more closely at the individual mouse data (Fig. S10†). This suggests that the interaction of SiO_2_ NPs with Bet v 1 exhibits a tendency to suppress IgE antibody levels. The determination of IgG antibody levels also revealed an increase in IgG1 and IgG2a antibody levels with Alhydrogel®-Bet v 1 (Fig. 7B, C and S11†). By investigating the averaged data there was no significant difference in the levels of IgG1 and IgG2a upon the interaction of SiO_2_ NPs with Bet v 1 or the BPE in comparison with the unconjugated allergenic compounds (Fig. 7B and C). However, if we examine the effects individually in mice, The interaction of SiO_2_ NPs with both Bet v 1 and the BPE demonstrated elevated IgG2a antibody levels, particularly in 3 of the 6 mice (Fig. 8), while the Bet v 1-specific IgG1 levels were not increased upon SiO_2_ NP interaction. In summary, when SiO_2_ NPs interact with the allergen, they tend to cause the Bet v 1-specific IgE antibody levels to decrease and the IgG2a antibody levels to increase. It has been demonstrated that allergen-specific IgG2 antibodies suppress IgE-mediated reactions.^66^ Moreover, the generation of IgG2a antibodies is associated with a Th1-skewing impact.^67^ Therefore, the increased IgG2a levels observed in mice treated with SiO_2_ NP–allergen conjugates would suggest the role of a Th1-mediated immune response.

Higher levels of allergen-specific IgE antibodies are in line with allergic sensitization. Obviously, SiO_2_ NPs are void of exacerbating allergic sensitization, as they induced a decrease in the Bet v 1-specific IgE antibody levels. There even may be a potential for SiO_2_ NPs to be used in AIT. However, this would be an object of further nanomedical engineering, for instance, by co-coupling SiO_2_ NPs with other immunostimulatory substances in addition to the allergen, as has been previously proposed.^13^ Using a non-viral plasmid DNA (pDNA)-encoded gene transfer approach, chitosan/IFN-γ pDNA nanoparticles have been shown to reduce allergic sensitization by lowering the levels of IgE antibodies specific for allergens with the help of *in situ* produced interferon (IFN)-γ, which in turn dampened airway inflammation and hyper responsiveness using an ovalbumin-sensitization model.^68^ Furthermore, it has previously been shown that a Japanese cedar pollen allergen conjugated with CpG oligodeoxynucleotides, the ligand of toll-like receptor 9, increases the allergen-specific Th1 responses in mice while suppressing IgE antibody and boosting IgG2a antibody levels.^69^ CpG ODN has been extensively studied as an adjuvant to boost the therapeutic benefits of AIT. Although it has been demonstrated that inducing both Th1 and Treg cells reduce allergy, it must be noted that it is preferable to elicit a Treg response rather than a Th1 response especially in AIT as the latter has been linked to unfavorable outcomes.^70^ In summary, the interaction between SiO_2_ NPs and allergens can inhibit allergic sensitization by eliciting a Th1-dominated response, which eliminates the possibility of any unfavorable effects related to allergic reactivity when applied in cosmetic products.

## Conclusions

There is growing evidence that engineered nanomaterials can either exacerbate or suppress allergic reactions; however, the contributing factors and molecular pathways affecting the allergic response under such conditions remain unknown. One such contributing factor would be the interaction of nanoparticles with the allergen forming an allergen corona, representing a genuine nanomaterial-specific molecular initiating event. The increased use of nanoparticles in cosmetic formulations enhances the chance for such a type of bio–nano interaction, affecting the structural integrity of the allergen, thereby modifying their sensitizing profile. In this study, we have determined the impact of allergen–nanoparticle interactions on allergic sensitization by studying the major molecular mechanisms that initiate an immune response. We observed enhanced uptake by macropinocytosis, proteolytic processing, and antigen presentation of the allergen together with the ability to boost IgG2a and diminish IgE antibody levels upon SiO_2_ NP interactions. All these events together imply the skewing of immune responses towards a Th1-dominated immune profile. As the immune deviation from Th2 to Th1 is considered as a protective immune response to allergens, this indicates that the non-covalent physical interaction of SiO_2_ NPs with allergens has the capacity to decrease allergic sensitization, as shown here for the case of birch pollen allergy, rather than exacerbate allergic sensitization. As a consequence, the use of SiO_2_ NPs in cosmetic products poses little risk, particularly in terms of the allergic response, and can, therefore, be regarded as safe. This mechanistic insight also signifies the potential application of SiO_2_ NPs in AIT. The immune deviation to Th1 and enhanced uptake of the allergen that was associated with the particle interaction are considered beneficial in AIT. Although the immune modulation from Th2 towards Th1 cells already represents an advantage for AIT, it would be optimal to elicit a Treg response. In this context, SiO_2_ NPs may benefit as an efficient nanocarrier platform for AIT when being associated with a non-particulate adjuvant that brings along a well-defined immunomodulatory property.

## Animals, materials and methods

### Synthesis and physicochemical characterization of nanoparticles

The microemulsion method was used to synthesize SiO_2_ NPs.^21^ The hydrodynamic size, polydispersity index and zeta potential of the synthesized particles were determined using NanoSight LM10 (Malvern Panalytical, Malvern, UK) and ZetaSizer Nano ZS (Malvern Panalytical) instruments. The primary size of particles was measured by transmission electron microscopy (EM 910, Zeiss, Oberkochen, Germany). The positive control for *in vivo* experiments (Alhydrogel®) was purchased from Brenntag, Germany and was characterized similarly.

### Nanoparticle–allergen conjugation

The choice of allergens for the study was the major birch pollen allergen, Bet v 1.0101 (Bet v 1). The recombinant allergen was expressed and purified in the laboratory following a previously published protocol.^71^ The preparation of birch pollen extract (BPE) is described in the ESI.† The nanoparticle–allergen conjugates were prepared by non-covalent adsorption, where 2 mg ml^−1^ of NPs were incubated with 160 μg ml^−1^ of Bet v 1 or 200 μg ml^−1^ of BPE for 17 hours at 4 °C while maintaining an isotonic environment and pH 7.4. The conjugation efficiency was determined by employing sodium dodecyl sulphate-polyacrylamide gel electrophoresis (SDS-PAGE), Bradford, and the bicinchoninic acid (BCA) assay as previously described.^21,72^

### Detection of endotoxin contamination

The endotoxin/LPS contamination in the particulate systems (SiO_2_ NPs and Alhydrogel®), Bet v 1, BPE and medium was determined by employing two different methods with varying principles. Initially the monocyte activation test (MAT) was employed which determines the changes in pro-inflammatory cytokine expression (IL-6 and TNF-α) in monocytes followed by incubation with samples. Monocytes were purified from peripheral blood mononuclear cells (PBMCs) using CD14 MicroBeads UltraPure (Miltenyi Biotech, Bergisch-Gladbach, Germany) by magnetic-activated cell sorting. The protocol from the manufacturer's instruction was followed without further modification. The purified monocytes were then seeded at a density of 3 × 10^5^ cells per ml (volume 100 μl) in a 96-well plate. This was followed by stimulation with SiO_2_ NPs (100 μg ml^−1^), Alhydrogel® (100 μg ml^−1^), Bet v 1 (10 μg ml^−1^) and BPE (100 μg ml^−1^). The samples were diluted in the cell culture medium to obtain a volume of 100 μl and incubated with the monocytes for 24 hours. The supernatants collected after the incubation were analyzed for the expression of IL-6 and TNF-α by enzyme-linked immunosorbent assay (ELISA) (Peprotech, London, UK). Additionally, HEK Blue™ hTLR4 LPS detection assay from Invitrogen (San Diego, CA, USA) was performed for further quantification of endotoxin. In this assay, a reporter cell line transfected with TLR4 and the secreted embryonic alkaline phosphatase (SEAP) reporter gene were used to detect the endotoxin levels. The assay was performed according to the manufacturer's instruction. The concentration of LPS in the samples was determined in both assays based on a standard LPS curve (1500 pg ml^−1^ to 1 pg ml^−1^). The LPS content represented in the monocyte activation test is the average of LPS concentrations measured in both IL-6 and TNF-α ELISA. Endotoxin-free water was used as the negative control and LPS from *E. coli* (100 ng ml^−1^) as the positive control in both the assays.

### Isolation of monocyte-derived dendritic cells (moDCs) and culturing

All studies involving human cells were conducted in accordance with the guidelines of the World Medical Association's Declaration of Helsinki. According to the national regulations, no additional approval by the local ethics committee was required in the case of anonymous blood cells discarded after plasmapheresis (buffy coats). Buffy coats from healthy (non-allergic) donors were kindly provided by the Salzburger Landesklinikum. Peripheral blood mononuclear cells (PBMCs) were isolated by density gradient centrifugation using histopaque-1077 (Sigma, St Louis, MO, USA). The adhesion method was used to separate the monocytes from the other PBMCs. Immature moDCs were generated from adherent monocytes by culturing 6 days in RPMI 1640 medium (Sigma) containing 10% heat-inactivated fetal calf serum (Biowest, Nuaillé, France), 2 mM l-glutamine (Sigma), 50 μM 2-mercaptoethanol (Sigma), 100 U mL^−1^ penicillin (Sigma) and 100 μg mL^−1^ streptomycin (Sigma) supplemented with 50 ng mL^−1^ granulocyte macrophage colony-stimulating factor (GM-CSF) and 50 ng mL^−1^ interleukin 4 (IL-4) (Life Technologies, Carlsbad, CA, USA). On day 3, one volume of fresh medium supplemented with 100 ng mL^−1^ GM-CSF and 100 ng mL^−1^ IL-4 was added. The detailed protocol was followed as previously described.^73^ For all the experiments, the cells were cultured at 37 °C by maintaining 5% CO_2_ and 95% humidity.

### Analysis of uptake

To determine the kinetics of uptake, moDCs were seeded in a 24-well plate at a density of 1 × 10^5^ cells per ml, stimulated with the samples (1 μg ml^−1^ of Bet v 1 and 1 μg ml^−1^ of Bet v 1 conjugated to 100 μg ml^−1^ of SiO_2_ NPs), and then incubated for different time periods (1, 2, 4, 6, 8 or 24 h). The viability of the cells on incubation with the samples was ensured before investigating the uptake (Fig. S12†). To assess the uptake of allergen by flow cytometry, Bet v 1 was fluorescently labelled with pHrodo™ Red succinimidyl ester (ThermoFisher Scientific, Waltham, MA, USA). The detailed protocol for fluorescent labelling of Bet v 1 is described in the ESI.† Four distinct inhibitors were used to study the mechanism of uptake: cytochalasin D (for macropinocytosis and phagocytosis), chlorpromazine hydrochloride (for clathrin-mediated endocytosis), filipin (for caveolin-dependent endocytosis), and rottlerin (for macropinocytosis) (Merck, Darmstadt, Germany). The detailed protocol together with the concentration of inhibitors, and the time of incubation can be found in the ESI.† To confirm the exocytosis of nanoparticles, the cells were incubated with 1 μg mL^−1^ of Bet v 1 conjugated to 100 μg mL^−1^ of SiO_2_ NPs for the defined time points, and washed three times to remove the excess nanoparticles which were present outside the cells/on the surface. The silica content of the NPs in the cells was determined by the blue silicomolybdic assay adapted for microtiter plates, as previously described.^74^ The adapted protocol can be found in the ESI.† Additionally, we measured the *in vitro* cellular uptake of nanoparticles using transmission electron microscopy (TEM).

### Transmission electron microscopy

moDCs were stimulated with 1 μg ml^−1^ of Bet v 1 conjugated to 100 μg ml^−1^ of SiO_2_ NPs for 4 hours. The cells were then centrifuged and washed three times with PBS to remove the excess particles. The cells were then placed in the sample holder for high pressure freeze fixation (HPF). A Leica Empact HPF device (Leica Microsystems, Wetzlar, Germany) was used for HPF at a cooling rate of about 12 000 °C per second and a pressure of at least 2040 bar. A Leica EM AFS (Leica Microsystems) was used to perform cryo-substitution at predetermined cycles using a substitution medium containing 2% osmium tetroxide (OsO_4_) and 0.05% uranyl acetate in anhydrous acetone. This was followed by three rounds of washing in anhydrous acetone and propylene oxide before being implanted in epoxy resin (medium grade; Agar Scientific, Stansted, Essex, UK). To guarantee that moDCs would sink and collect at the concave bottom of the capsules for subsequent processing, the samples were embedded in Beem® capsules (Agar Scientific). The samples were then polymerized at 70 °C for 24 hours. A Leica UC7 ultramicrotome (Leica microsystems) was used to perform ultra-thin sectioning (approx. 70 nm) and the sections were placed on Formvar-coated copper grids for imaging. moDCs were imaged using a Zeiss LEO 912 AB TEM with an in-column Omega energy filter at an accelerating voltage of 80 kV. A Tröndle TRS Sharp Eye bottom-mounted 2 K CCD camera was used to capture TEM pictures and the process was recorded and controlled using iTEM 5.0 software (Olympus).^75^

### Proteolytic degradation of allergens

The samples (Bet v 1/BPE conjugated to SiO_2_ NPs and Bet v 1/BPE only) containing 20 μM equivalent Bet v 1 were incubated in 0.1 M sodium acetate pH 5, 0.1 M sodium chloride, 5 mM EDTA and 2 mM DTT with 1 μM of recombinant human cathepsin S (rCathepsin S) for 0, 0.5, 1, 3, 6, 12, 24 and 48 h at 37 °C. rCathepsin S was produced and purified in accordance with previously described procedures.^46^ At the end of each incubation, the enzymatic degradation was stopped by the addition of 50 μM E64 (cathepsin S inhibitor). The intact protein at different time points was analyzed by SDS-PAGE and quantitatively determined using Image Lab 4.01 software (Bio-Rad). The percentage of proteolytic stability was calculated based on the 0 hour time point. For the BPE, only the intensity of Bet v 1 bands was taken into consideration.

### Antigen presentation of allergens

Murine bone marrow-derived dendritic cells (BMDCs) from BALB/c mice were exposed to the conjugated and unconjugated samples at a concentration of 1 μg ml^−1^ of Bet v 1 or 10 μg ml^−1^ of BPE conjugated to 100 μg ml^−1^ of SiO_2_ NPs for various time periods. After incubation, BMDCs were washed and co-cultured with CD4^+^ T cell hybridoma cells specific for the immunodominant epitope of Bet v 1 (amino acids 142–153) for 16 hours. The supernatants were then collected, and the IL-2 release was quantified by ELISA (ELISA MAX™ standard set mouse IL-2, Biolegend, San Diego, CA, USA).

### Flow cytometry

The surface marker expression of moDCs was analyzed using flow cytometry. moDCs were seeded in a 24-well plate at a density of 1 × 10^5^ cells per ml, and stimulated with the samples including 1 μg ml^−1^ of Bet v 1 or 10 μg ml^−1^ of BPE conjugated to 100 μg ml^−1^ of SiO_2_ NPs for 24 hours. The cells were then washed and stained with α-HLA-DR APC (Invitrogen, Waltham, MA, USA), Fixable Viability Dye eFluor 506 (eBioscience, Waltham, MA, USA), α-CD1a BV421 (Biolegend), α-CD86 PE (eBioscience), α-CD40 FITC (Biolegend), α-CD80 APC-H7 (BD Biosciences, Heidelberg, Germany) and α-CD83 PE-Cy™7 (BD Biosciences) for 30 minutes at 4 °C in the dark. The cells were then fixed with 4% PFA, then suspended in FACS buffer containing PBS and 3 mM EDTA, acquired using an FACS Canto II flow cytometer (BD Biosciences) and analysed using FlowJo X 10.0.7r2 software (BD Biosciences). The cells were washed twice with cold PBS in between the consecutive steps. LPS (100 ng ml^−1^) was used as the positive control and unstimulated cells were used as the negative control. The gating strategy used for the analysis and the FMO controls are shown in Fig. S13.†

### Cyto/chemokine multiplexing

45-Plex Human Procarta-Plex™ (ThermoFisher, Waltham, Massachusetts, USA) was used to quantify the release of cytokines and chemokines from moDCs stimulated with samples in accordance with the manufacturer's recommendations. A magnetic bead mixture was prepared, washed with wash buffer (PBS, 0.05% Tween-20) and suspended in assay buffer (PBS, 0.05% Tween-20, 1% FCS) (Biowest, Nuaillé, France). 8.34 μl of the beads were then pipetted to each well of a 96-well V-bottom plate followed by the addition of 15 μl of samples and standards. The resulting mixtures were incubated at 4 °C in an orbital shaker (500 rpm) protected from light overnight. The wells were then washed and incubated with 15 μl of detection antibody solution for 30 minutes in the dark. Each well received 20 μl of streptavidin–PE solution (1 : 1 in assay buffer) and it was left at room temperature for 30 minutes in the dark. The samples were then suspended in drive fluid and measured in a Luminex Magpix Multiplex machine (ThermoFisher). Procarta Plex Analyst software 1.0 (ThermoFisher) was used to process the data.

### *In vivo* experiments

Female BALB/c mice, aged 6–8 weeks, were purchased from Janvier (Le Genest-Saint-Isle, France), and taken care in a pathogen-free environment at the animal facility of the University of Salzburg in accordance with local regulations. The study used female mice since it has been observed that they exhibit more severe allergic airway inflammation when compared to male mice.^76^ For studying immunogenicity, the mice were treated with the samples (6.5 μg of Bet v 1, 6.5 μg of Bet v 1 with 250 μg of SiO_2_ NPs, 6.5 μg of Bet v 1 with 250 μg of Alhydrogel®, 65 μg of BPE, 65 μg of BPE with 250 μg of SiO_2_ NPs, 250 μg of SiO_2_ NPs, 250 μg of Alhydrogel® and PBS) on days 0, 14, 28 and 42 by subcutaneous injection.^28^ Blood samples were taken at day 56 and stored at −20 °C. Bet v 1-specific IgG subclasses were measured by direct ELISA using rat anti-mouse IgG1-HRP and rat anti-mouse IgG2a-HRP antibody (SouthernBiotech, AL, US), and Bet v 1-specific IgE in the sera was determined by the murine rat basophil leukaemia cell assay (muRBL). The detailed protocols are described in the ESI.†*In vivo* experiments were performed according to national guidelines approved by the Austrian Federal Ministry (BMBWF - V/3b; approval number 2021-0.118.574).

## Author contributions

Conceptualization: L. J., L. A., and M. H.; investigation: L. J., B. P., H. D., C. C., M. W., A. A., and M. G.; methodology: J. H., L. A., L. P., A. A., and H. D.; formal analysis: L. J., B. P., H. D., C. C., M. G., and M. H.; visualization: L. J., B. P., S. H., and N. H.; funding acquisition and supervision: F. F., J. H., A. D. and M. H.; writing–original draft preparation: L. J.; and writing–review and editing: L. J., L. A., F. F., J. H., A. D. and M. H. All authors have read and agreed to the published version of the manuscript.

## Conflicts of interest

There are no conflicts to declare.

## Supplementary Material

NR-015-D2NR05181H-s001
