# Supplementary material for: Mechanistic insights into silica nanoparticle–allergen interactions on antigen presenting cell function in the context of allergic reactions
Source: Nanoscale. 2023 Jan 11;15(5):2262–75. doi: 10.1039/d2nr05181h (PMC9893438; doi:10.1039/d2nr05181h)
Supplement: NR-015-D2NR05181H-s001 [file NR-015-D2NR05181H-s001.pdf]

Electronic Supplementary Material (ESI) for Nanoscale.
This journal is © The Royal Society of Chemistry 2023

Mechanistic insights into silica nanoparticle-allergen interaction on antigen presenting cell function in the context of allergic reactions

Litty Johnson, Lorenz Aglas, Benjamin Punz, Hieu-Hoa Dang, Constantin Christ, Lisa Pointner, Mario Wenger, Norbert Hofstaetter, Sabine Hofer, Mark Geppert, Ancuela Andosch, Fatima Ferreira, Jutta Horejs-Hoeck, Albert Duschl and Martin Himly*

Electronic Supplementary Information

Methods in detail

Preparation of birch pollen extract (BPE)

1.8 g of commercial birch pollen (*Betula pendula*, Thermo Fisher Scientific) was suspended in 1 ml of PBS in a low-binding Eppendorf reaction tube. After mixing thoroughly the suspension was incubated in a shaker for 1 hour at room temperature, followed by centrifugation at 18,000* *g* for 15 min. The supernatant collected was filtered through a 0.22 µm filter and stored at -20°C. The filtrate was thoroughly mixed with a 1 ml pipette, and the total protein concentration was measured using Bradford, and the protein profiles were characterized by SDS-PAGE and mass spectrometry.

Fluorescent labelling of Bet v 1

1 mg of Bet v 1 was incubated with 10-fold molar excess of pHrodo™ red succinimidyl ester (Thermofischer Scientific, Waltham, Massachusetts, United States) in 1 M $NaHCO_3$ buffer (pH 8.8) at room temperature for 1.5 hours. The total reaction volume was 500 µl. The labelled fraction of protein was separated from the excess fluorescent label reagent by size exclusion chromatography using Cytiva NAP-5 column (Sigma-Aldrich) and 5 mM sodium phosphate buffer (pH 7.4) as the eluent. The protein content in the labelled fraction was determined using the Nanodrop™ NO-1000 spectrophotometer (Thermofischer Scientific).

Mechanism of uptake-inhibitors

moDCs were seeded at a density of $1*10^5$ cells/ml in a 24-well plate and pre-incubated with the defined concentration of inhibitors for desired times (Table S1). The concentration was chosen based on a standard titration of different concentrations of

inhibitors to achieve >85% viability of cells. The pre-incubation was followed by the stimulation of moDCs with the samples for 24 hours.

Table S1. List of the inhibitors used with the concentration and pre-incubation time

Inhibitor	Mechanism/Inhibition	Pre-incubation time	Concentration (in well)
Cytochalasin D (CytoD)	Macropinocytosis/phagocytosis	90 min	2 μM
Chlorpromazine hydrochloride (CPZ)	Clathrin-mediated endocytosis	30 min	20 μM
Filipin	Caveolin-dependent endocytosis	30 min	1 μM
Rottlerin	Macropinocytosis	30 min	10 μM

Silicomolybdic assay

The cell samples at different time points were digested with 0.5 M NaOH for 1 hour at 95ºC, followed by dilution in water (1:15). 300 μl of the diluted samples were then incubated with 30 μl of solution containing 0.016 M ammonium molybdate tetrahydrate and 0.7 M hydrochloric acid in pure water for 10 min. In the next step, 150 μl of solution containing 0.22 M oxalic acid, 0.019 M 4-methylaminophenol sulphate, 0.033 M anhydrous sodium sulphite and 1.8 M concentrated sulphuric acid in pure water was added and incubated for 2 hours in Eppendorf tubes. The absorbance of the samples was then measured at 810 nm and the silica content was quantified based on known concentration of silica standards.

Direct ELISA for determining Bet v 1-specific IgG

2 μg/ml of Bet v 1 was coated onto 96-well plates through passive adsorption and incubated overnight at 4ºC. This was followed by washing the plates and blocking unbound sites with 1% BSA in PBS. The sera from the mice were then incubated for 2 hours at room temperature followed by incubation with the detection antibody (anti-IgG1-HRP and anti-IgG2a-HRP) for 2 hours. The absorbance was measured at 450 nm by the addition of substrate and stop solution. The AUC values were calculated from the graphs presenting absorbance values *vs*. log dilutions using the trapezoid rule employing the GraphPad Prism 9.4. For IgG1 the AUC was determined for serum dilutions ranging from 200 to 102400, whereas for IgG2a the serum dilutions were

ranging from 50 to 1600 (Figure S11). The AUC was computed using the lowest value as baseline.

muRBL assay for IgE estimation

Rat basophilic leukaemia cells (RBL-2H3) transfected with murine high-affinity IgE receptor (FcεRI) were sensitized with sera from the mice overnight at 37 °C and maintaining 5% CO_2. The washed cells (in tyrodes buffer) were then incubated with Bet v 1 in serial dilutions (1000 µg/ml to 0.0001 µg/ml). The fluorogenic substrate 4-methyl umbelliferyl-N-acetyl-glucosaminide (Sigma-Aldrich, St. Louis, MO, USA) was then added to the collected supernatants after the incubation period. Upon release of β-hexosaminidase into the supernatant it cleaves the fluorogenic substrate, leading to fluorescence. The reaction was terminated after 1 hour and the fluorescence intensity was measured at an excitation wavelength of 360 nm and emission at 440 nm. The percentage of release was calculated by comparing it with the maximum release attained with 10% Triton X 100. The results were expressed as area under the curve (AUC).

Supplementary data

Table S2: Physicochemical characterization of the synthesized SiO2 NPs and Alhydrogel® by measurement of hydrodynamic size, poly-dispersity index and zeta potential by DLS (NP concentration of 0.1 mg/ml) and NTA (NP concentration of 0.02 mg/ml).

Sample	Technique	Number mean diameter (nm)	PDI	Size (Z average) (nm)	Zeta Potential (mV)
SiO_2 NPs	DLS	100.3±3.4	0.025	120.2±1.2	-38.9±2.8
SiO_2 NPs	NTA	102.4±39.3	-	-	-
Alhydrogel®	DLS	585.9±174.2	0.345	1082.0±63.4	+18.0±1.5

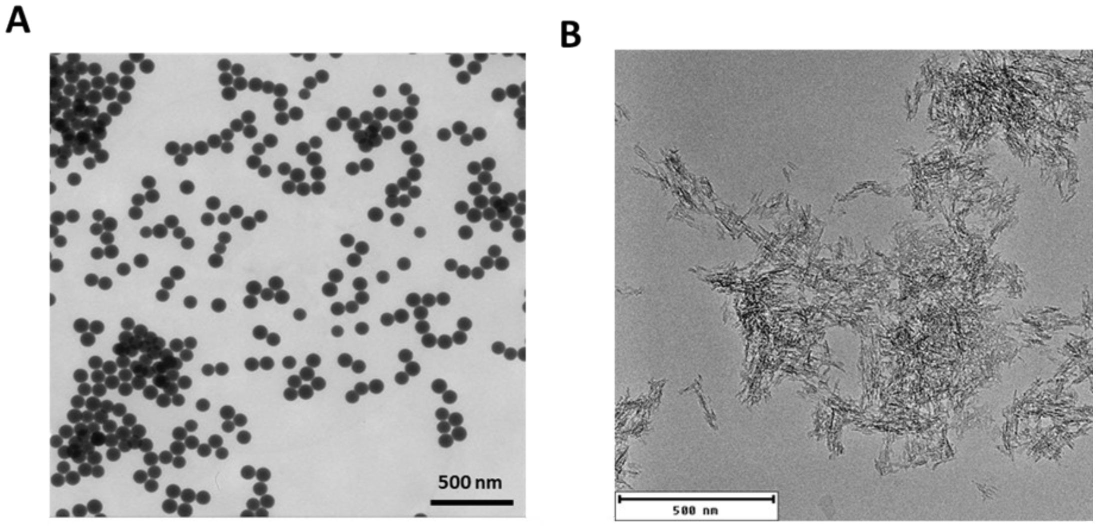

Figure S1. Characterization of the particulate systems by determining the morphology and primary size of particles. Transmission electron microscopy (TEM) image of synthesized SiO_2 NPs **(A)** and Alhydrogel® **(B)**

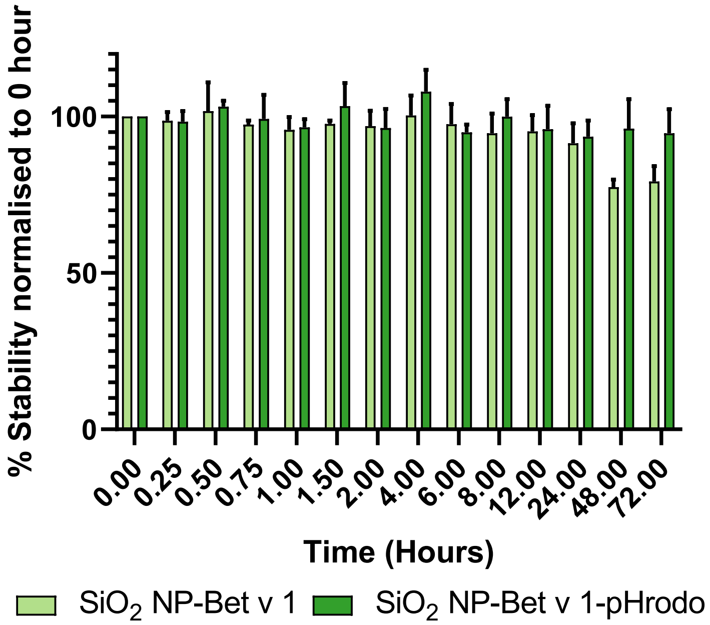

Figure S2. The suspension stability of nanoparticle-allergen conjugates in moDC medium at different time points, determined by silicomolybdic assay. A concentration of 1 µg/ml of allergen bound to 100 µg/ml of SiO_2 NP in a volume of 5 ml of moDC medium at 37°C was used to assess the stability.

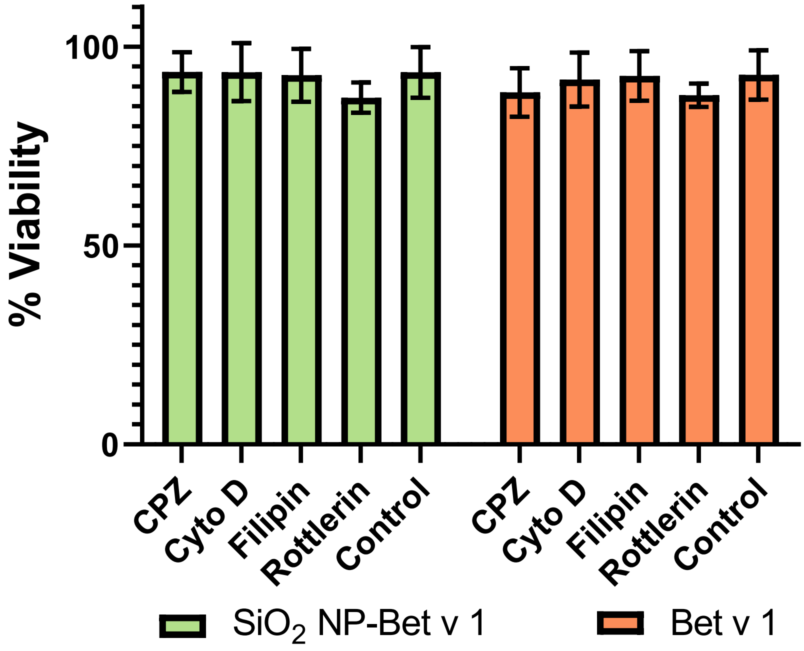

Figure S3. Viability of moDCs after incubation with the inhibitors for the desired time of 24 hours. The percentage viability was calculated based on the comparison to unstimulated cell control

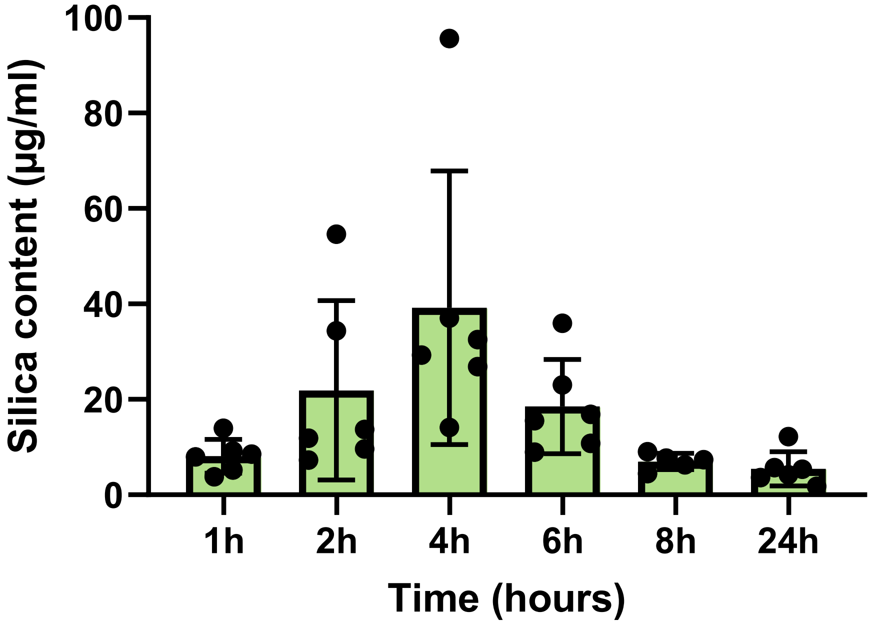

Figure S4. Exocytosis of SiO_2 NP from moDCs determined by measuring the intracellular silica content.

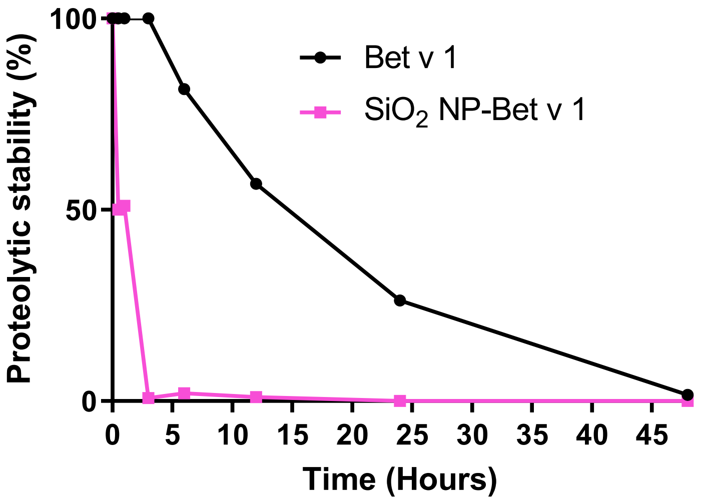

Figure S5: Proteolytic degradation of allergen using the microsomal extracts from JAWS II determined by endolysosomal degradation assay.

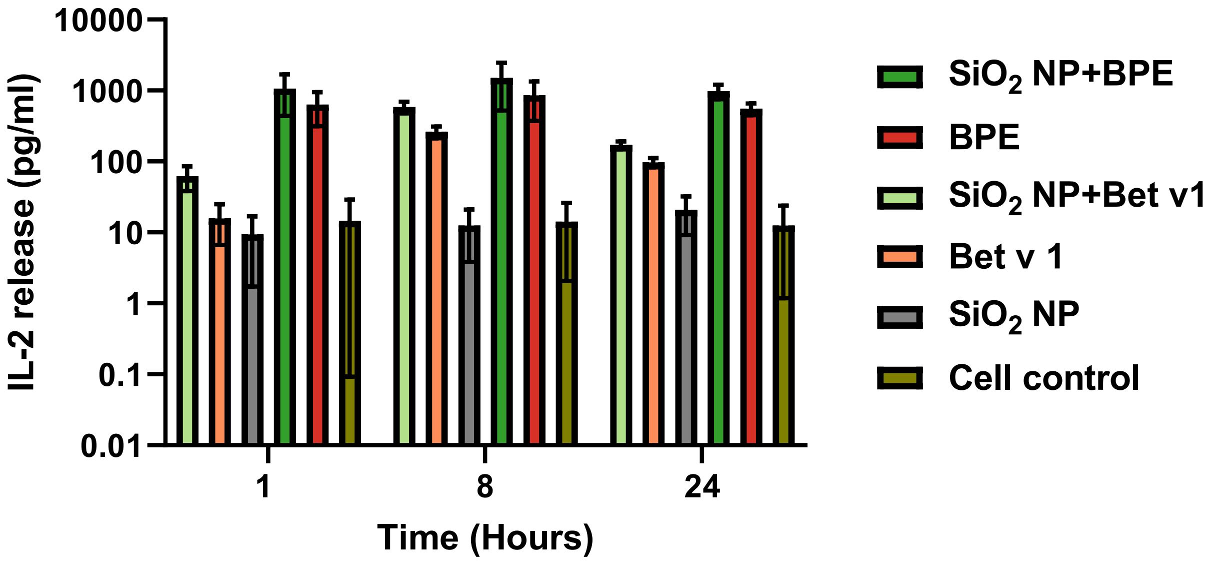

Figure S6. Activation of naïve T cells upon presentation of allergen by APCs measured by the IL-2 release.

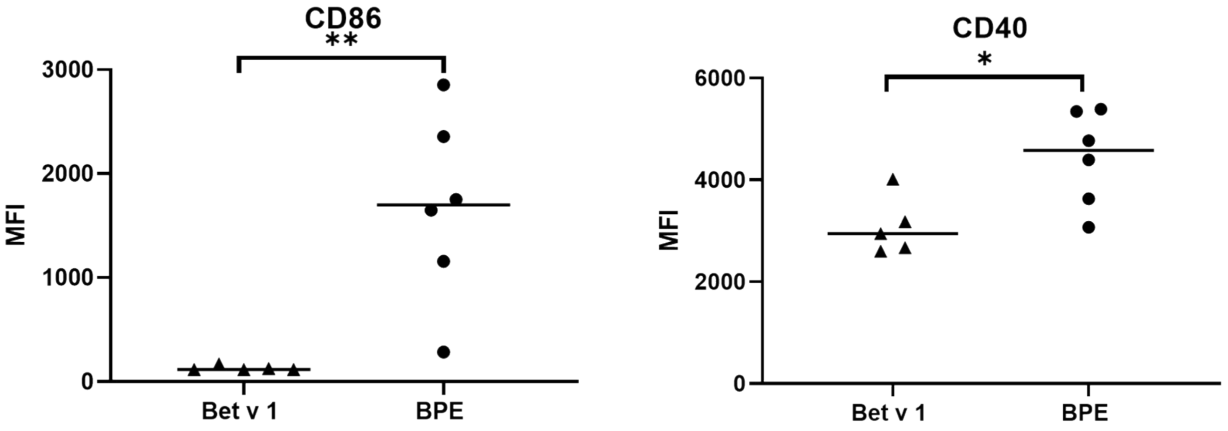

Figure S7. Differences in the expression of DC maturation markers, CD86 and CD40 in moDCs when stimulated with Bet v 1 and BPE. MFI, mean fluorescence intensity calculated by employing the FlowJo software (BD Biosciences).

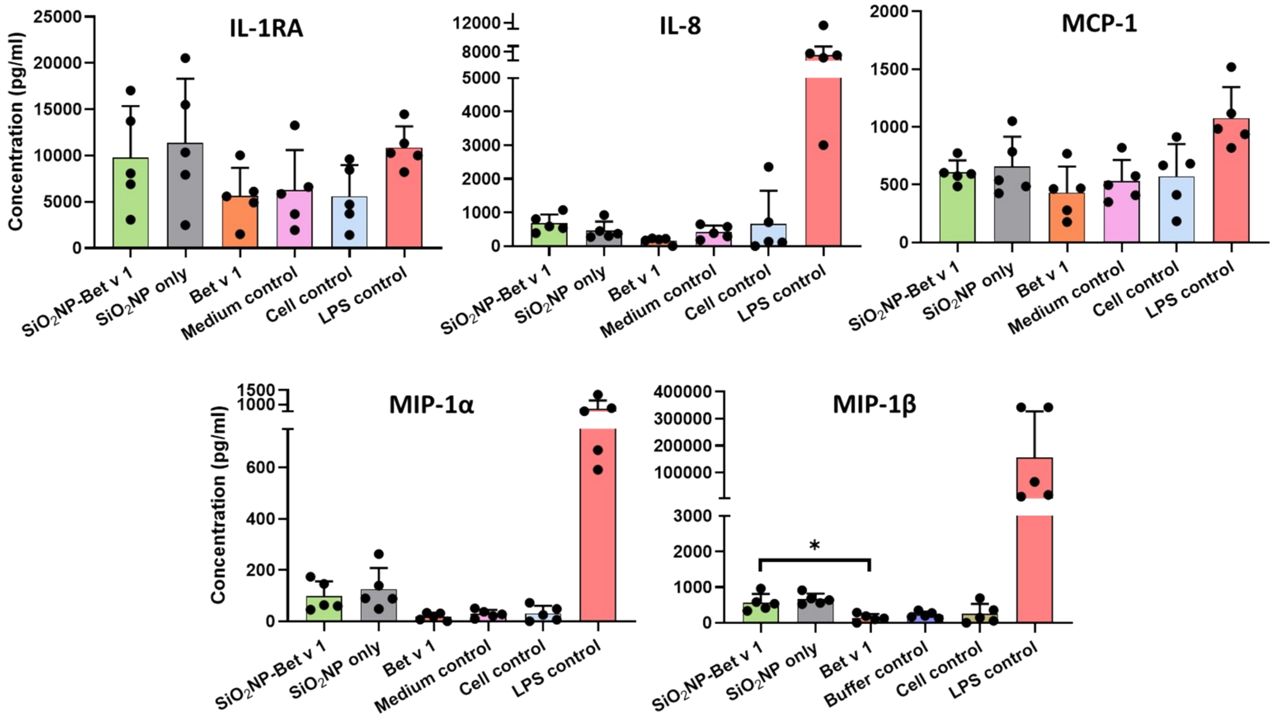

Figure S8. SiO_2 NP interaction with Bet v 1 does not influence the cytokine secretion in moDCs. The expression of 45 cytokines was analyzed from the supernatant after stimulation of moDCs with the samples for 24 hours using human Procarta-Plex^{TM} (ThermoFisher Scientific). 100 ng/ml of LPS was used as the positive control.

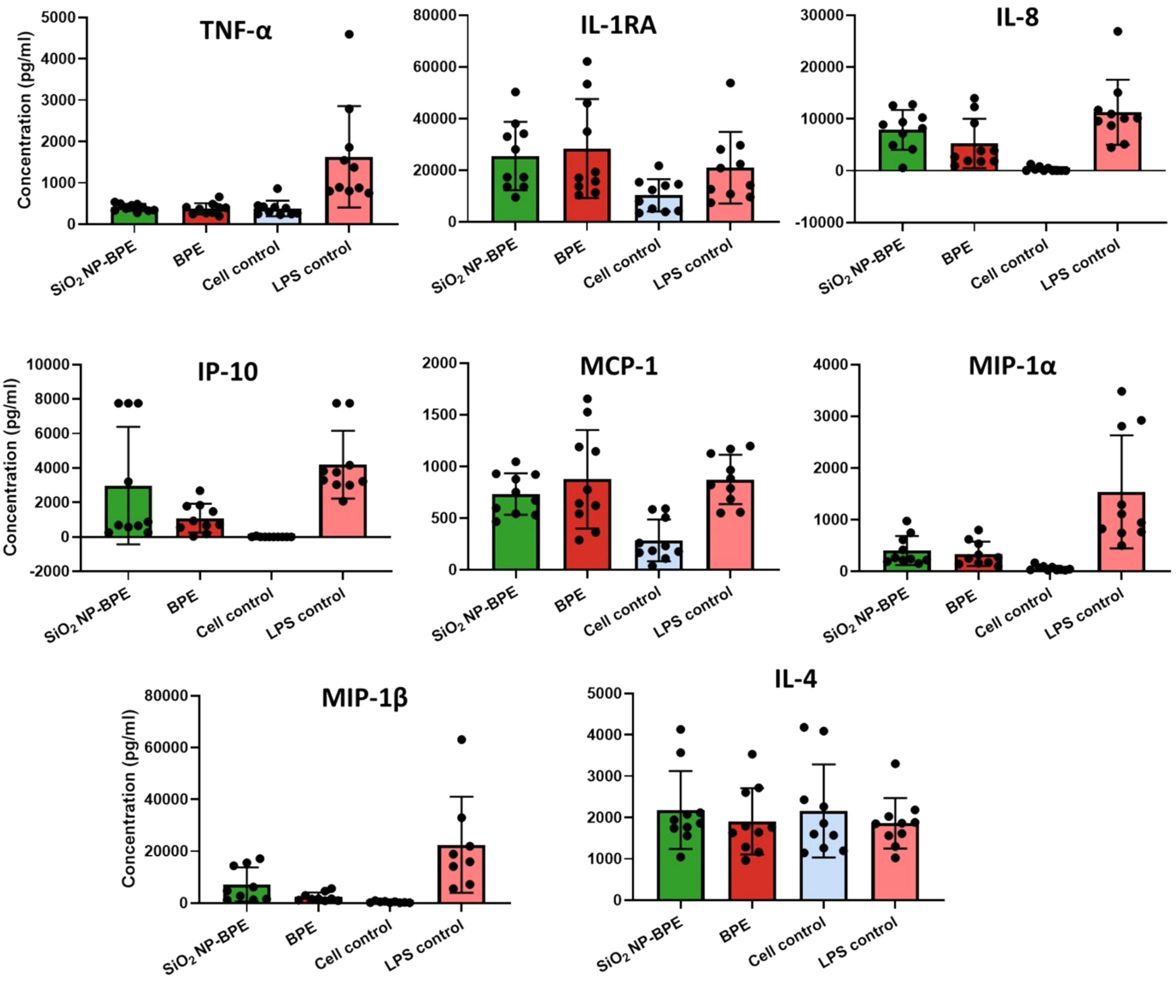

Figure S9. SiO_2 NP interaction with BPE does not influence the cytokine secretion in moDCs. The expression of 45 cytokines was analyzed from the supernatant after stimulation of moDCs with the samples for 24 hours using human Procarta-Plex^{TM} (ThermoFisher Scientific). 100 ng/ml of LPS was used as the positive control.

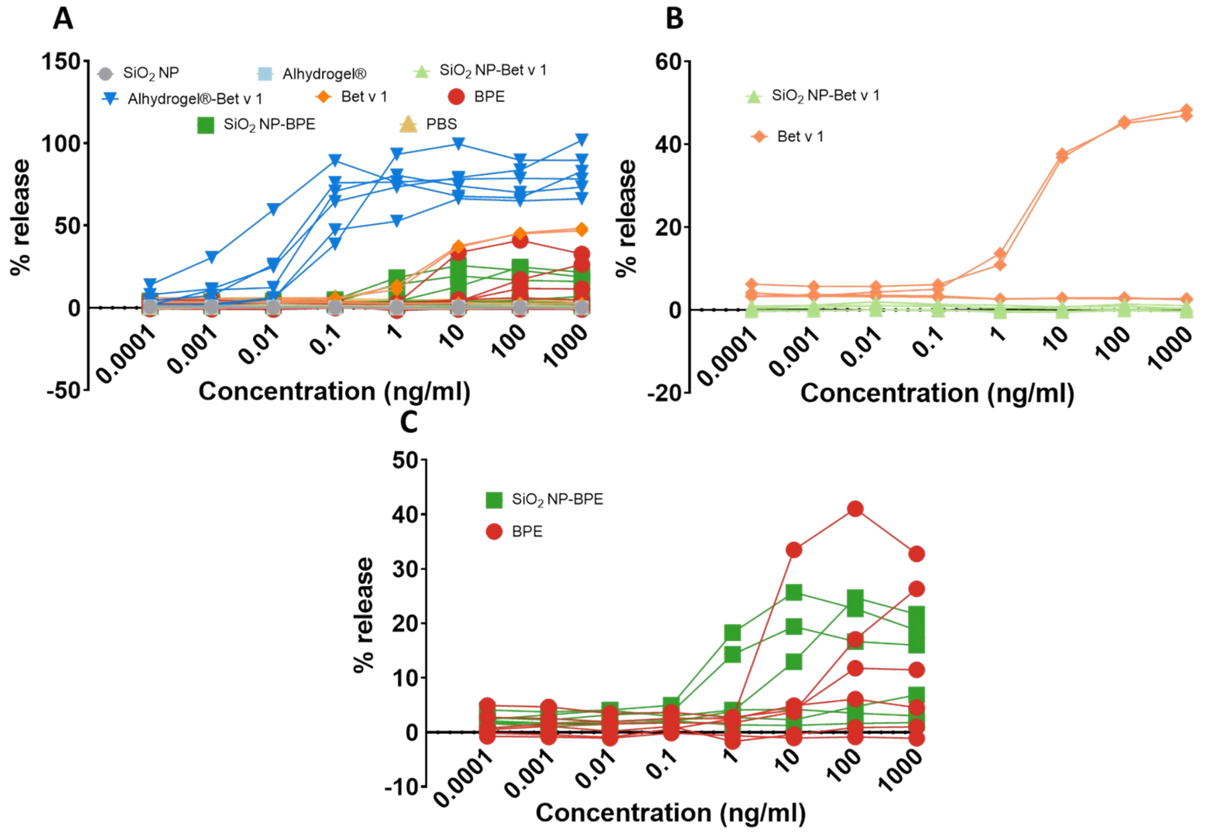

Figure S10. Quantification of the biologically active Bet v 1-specific IgE antibody levels by measuring the β-hexosaminidase mediator release. Degranulation of muRBL cells loaded with the sera of mice subcutaneously injected with the samples were measured upon induction with Bet v 1. **A)** Percentage mediator release of all samples, comparison of percentage release of **B)** SiO_2 NP-Bet v 1 *vs.* Bet v 1 **C)** SiO_2 NP-BPE *vs*. BPE.

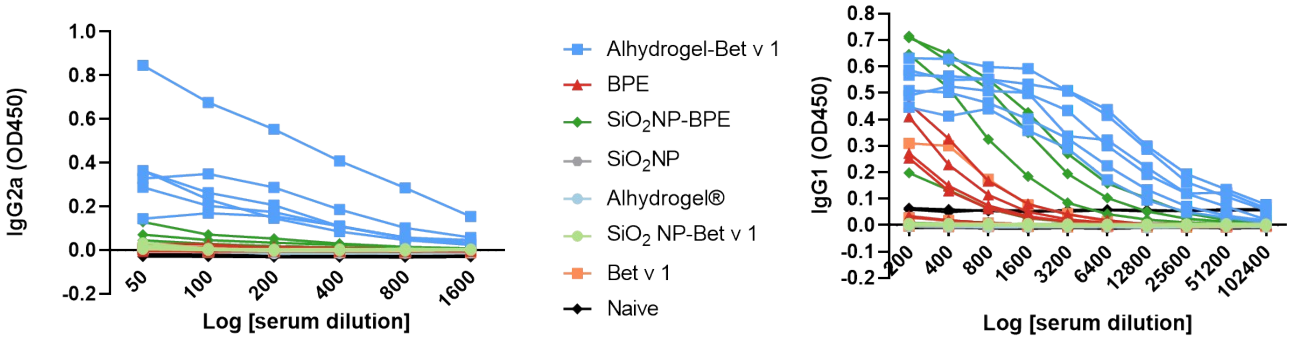

Figure S11. Raw absorbance values of the IgG2a and IgG1 antibody levels with different serum dilutions.

Table S3: Endpoint titers for IgG2a and IgG1 of Alhydrogel-Bet v 1

Mice	Endpoint titer IgG2a	Endpoint titer IgG1
M1	0.000046	0.0000007
M2	0.000266	0.0000146
M3	0.000392	0.0000026
M4	0.000321	0.0000130
M5	0.000189	0.0000012
M6	0.000252	0.0000020

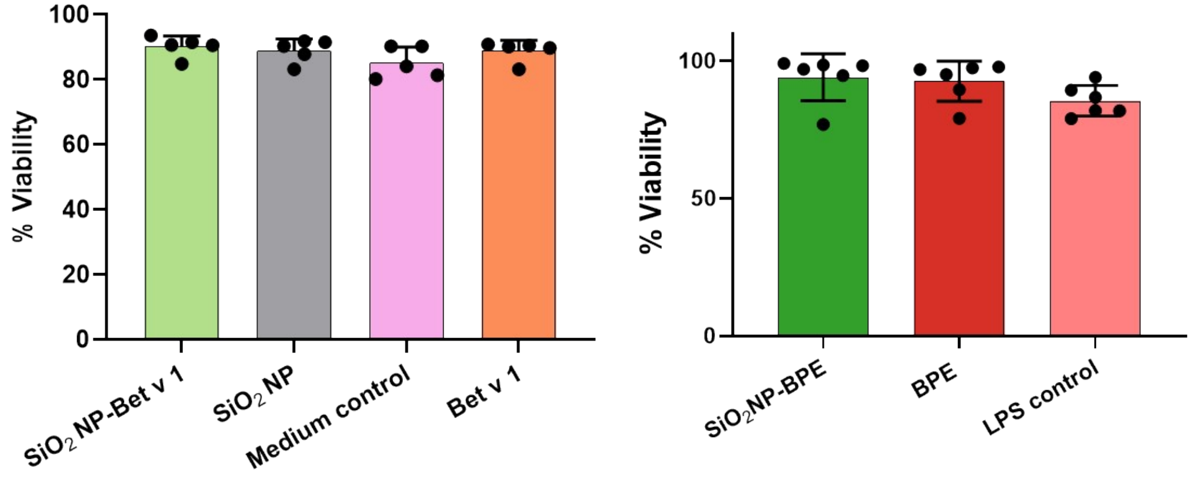

Figure S12. Viability of moDCs after incubation with the samples for 24 hours. The percentage viability was calculated based on the comparison to unstimulated cell control.

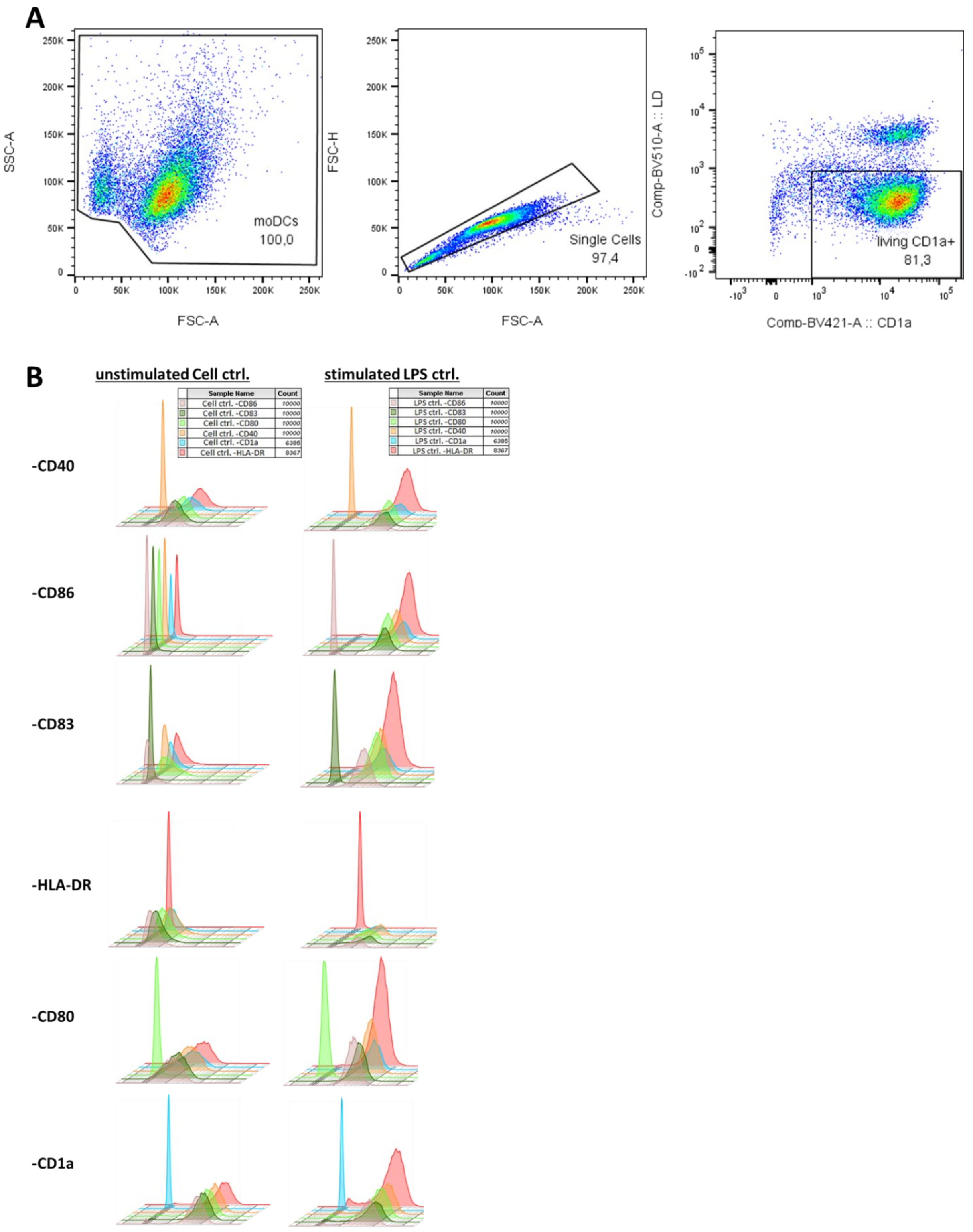

Figure S13. A) Gating strategy for moDCs generated from human buffy coats. MoDCs were gated based on singlets and CD1a^{+}-live cells **B)** Fluorescence minus one (FMO) controls of unstimulated *vs.* LPS-stimulated moDCs. One dye was removed each from the mix control for measuring the potential interference. CD40- FITC-A; CD86- PE-A; CD83- PE-Cy7-A; HLA-DR- APC-A; CD80- APC-Cy7-A; CD1a- BV421-A.
